# Long noncoding RNA hotair mediated angiogenesis in nasopharyngeal carcinoma by direct and indirect signaling pathways

**DOI:** 10.18632/oncotarget.6731

**Published:** 2015-12-22

**Authors:** Wei-ming Fu, Ying-fei Lu, Bao-guang Hu, Wei-cheng Liang, Xiao Zhu, Hai-di Yang, Gang Li, Jin-fang Zhang

**Affiliations:** ^1^ School of Medicine, South China University of Technology, Guangzhou 511458, P.R. China; ^2^ Guangzhou Institute of Advanced Technology, Chinese Academy of Sciences, Guangzhou 511458, P.R. China; ^3^ Department of Orthopaedics and Traumatology, The Chinese University of Hong Kong, Prince of Wales Hospital, Shatin, Hong Kong, P.R. China; ^4^ Department of Gastrointestinal Surgery, The Affiliated Hospital of Binzhou Medical University, Binzhou, Shandong, P.R. China; ^5^ School of Biomedical Sciences, Faculty of Medicine, The Chinese University of Hong Kong, Hong Kong, P. R. China; ^6^ Guangdong Province Key Laboratory of Medical Molecular Diagnosis, Guangdong Medical College, Dong guan, 523808, P.R. China; ^7^ Department of Otolaryngology, Sun Yat-Sen Memorial Hospital, Sun Yat-Sen University, Guangzhou, P.R. China; ^8^ Shenzhen Research Institute, The Chinese University of Hong Kong, Shenzhen, P.R. China

**Keywords:** Hotair, angiogenesis, nasopharyngeal carcinoma, GRP78

## Abstract

Nasopharyngeal carcinoma (NPC), as a unique head and neck cancer type, is particularly prevalent in certain geographic areas such as eastern Asia. Until now, the therapeutic options have been restricted mainly to radiotherapy or chemotherapy. However, the clinical treatment effect remains unsatisfactory even if the combined radio-chemotherapies. Therefore, it is urgently needed to develop effective novel therapies against NPC. In this study, we discovered that lncRNA Hotair was extremely abundant in NPC cells and clinical NPC samples. Further studies showed that Hotair knockdown significantly attenuated both *in vitro* and *in vivo* tumor cell growth and angiogenesis. Our study also demonstrated that Hotair promoted angiogenesis through directly activating the transcription of angiogenic factor VEGFA as well as through GRP78-mediated upregulation of VEGFA and Ang2 expression. Therefore, Hotair may serve as a promising diagnostic marker and therapeutic target for NPC patients.

## INTRODUCTION

Nasopharyngeal carcinoma (NPC) is an aggressive squamous cell carcinoma resides in nasopharynx, which is most common in Southeast Asia and North Africa. Besides genetic susceptibility and environmental factors, the Epstein-Barr virus (EBV) infection plays an important role in NPC pathogenesis which makes it unique among various head and neck cancer types. Until now, the therapeutic options have been limited mainly to mono-radiotherapy or concurrent adjuvant chemotherapy [1]. Considering that NPC is highly malignant, invasive and metastatic, the prognosis remains poor and the relapse rates reach as high as 82% in spite of combined radiation and chemotherapy treatment [2]. Furthermore, in terms of that most NPC patients is diagnosed at an advanced stage, the therapeutic approaches remain limited and the clinical outcome is still unsatisfactory [3]. Therefore, it is urgently needed to develop effective diagnostic biomarkers as well as novel therapeutic targets for NPC patients.

Long noncoding RNAs (lncRNAs) are non-protein-coding transcripts more than 200 nucleotides in length that are ubiquitous in mammalian genomes [4–5]. With advances in science and technology, lncRNAs have been identified as novel regulators of the transcriptional and epigenetic network. HOX antisense intergenic RNA (Hotair), a lncRNA initially identified in breast cancer, mediated tumorigenesis and metastasis in a variety of carcinomas [6–9]. The aberrantly up-regulated Hotair was also detected in several other tumors, such as colorectal cancer [10], cervical cancer [11], bladder cancer [12], hepatocellular carcinoma [13], gastrointestinal stromal tumors [14], and pancreatic cancer [15], and its expression positively correlates with poor prognosis, tumor progression and recurrence in these cancers. Although Hotair serves as an independent prognostic marker for NPC progression and survival [16], the underlying mechanism still remains elusive.

In the present study, Hotair was found to be markedly up-regulated in NPC cells and specimens. The subsequent functional studies revealed that Hotair knockdown suppressed cell proliferation and angiogenesis *in vitro* and *in vivo*. Moreover, the underlying mechanism of Hotair knockdown-suppressed angiogenesis was identified, namely, by which siHotair directly inactivated VEGFA transcriptional activity as well as suppressed the expression of glucose regulated protein 78 (GRP78). Collectively, our study dissected a novel pro-angiogenesis function of Hotair in NPC and it might serve as a promising diagnostic and therapeutic target for NPC patients.

## RESULTS

### Hotair was especially up-regulated in NPC cell lines and tissues

**Figure 1:**
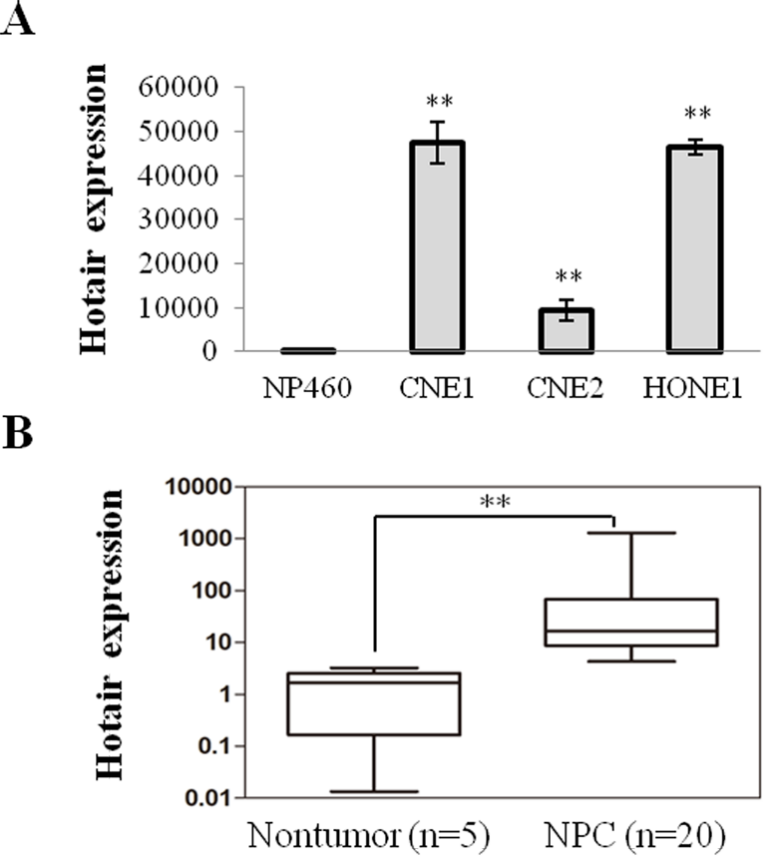
Hotair was up-regulated in NPC cells and specimens (**A**) Hotair was up-regulated in a panel of NPC cells. (**B**) increased expression of Hotair was found in NPC specimens. *P < 0.05; **P < 0.01.

Hotair exhibits the pro-oncogenic activity in multiple cancers and our results revealed that Hotair was considerably up-regulated in NPC cells compared with the immortalized Nasopharyngeal cell line NP460 (Figure 1A). Furthermore, it was also significantly increased in NPC tissues compared to normal ones (Figure 1B). Therefore, consistent with previous studies, increased Hotair expression frequently occurs in NPC, suggesting that it may mediate tumor development and progression.

### Hotair mediated cell growth and angiogenesis in NPC cells *in vitro* and *in vivo*

**Figure 2:**
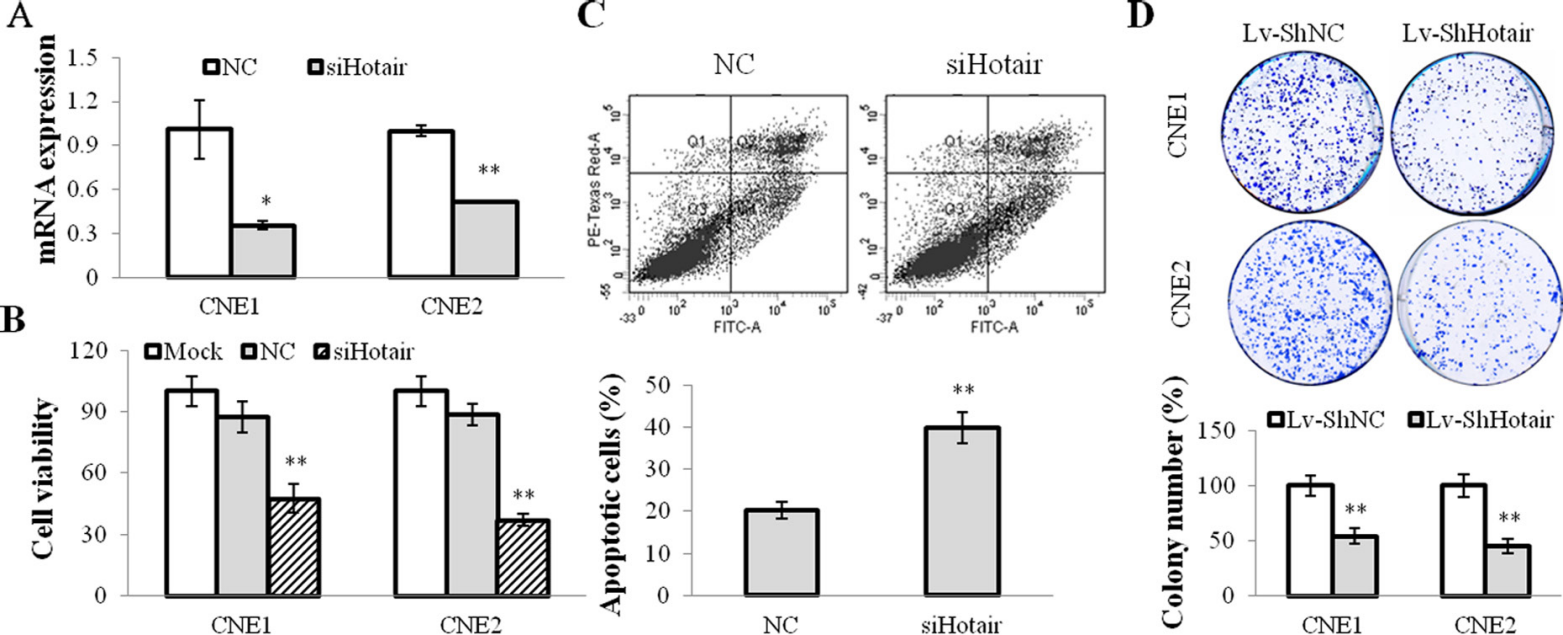
Hotair mediated cell growth in NPC cells (**A**) Hotair expression was suppressed by siHotair in NPC cells. (**B** & **C**) siHotair suppressed cell viabilities (B) and induced apoptosis in CNE1 cells (C). Top, the representative result of one such assay; and bottom, mean ± SD of three independent experiments. (**D**) the Lv-ShHotair infected NPC cells formed significantly less colonies, as compared to the Lv-ShNC infected cells. *P < 0.05; **P < 0.01.

To explore the function of Hotair on tumorigenesis, the specific siRNA against Hotair (siHotair) was transfected into NPC cells and the cell viabilities were detected. The results displayed that Hotair expression was suppressed by siHotair (Figure 2A) and a significant suppressive effect on cell viability was observed in CNE1 and CNE2 cells (Figure 2B). In addition, the subsequent apoptosis assay revealed that more apoptotic cells were induced by siHotair in CNE1 cells (Figure 2C). Moreover, Lv-ShHotair infected NPC cells formed much fewer colonies compared with those obtained with Lv-ShNC-infected cells (Figure 2D). Collectively, these data suggested that Hotair knockdown suppressed cell viability and induced apoptosis in NPC cells.

**Figure 3:**
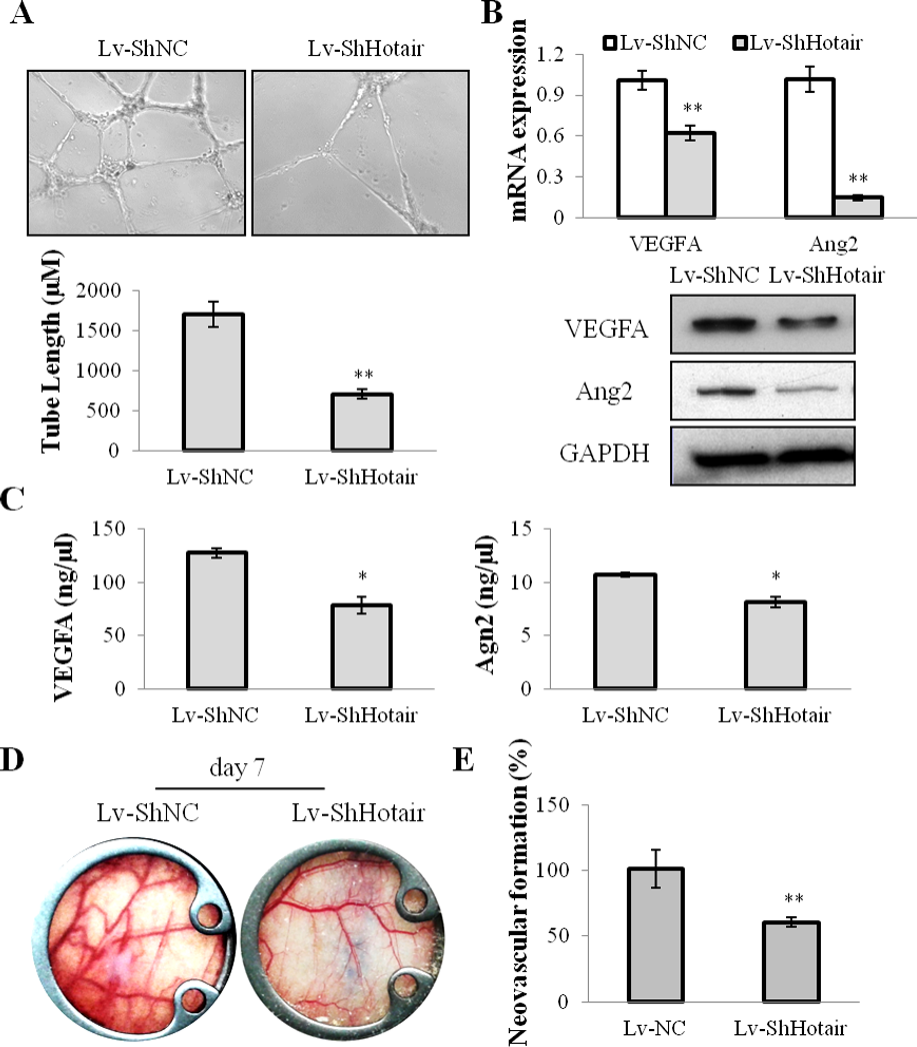
Hotair mediated angiogenesis in NPC cells (**A**) the ability of endothelial cell tube formation was suppressed in the Lv-ShHotair-infected condition medium, as compared to the Lv-ShNC infected condition medium. (**B**) the angiogenic growth factors VEGFA and Ang2 were down-regulated in the Lv-ShHotair infected CNE1 cells at mRNA and protein levels. (**C**) the secretion of VEGFA and Ang2 was reduced in the Lv-ShHotair infected CNE1 cells. (**D** & **E**) the promoting effect of CNE1 cells on neovascularization was significantly inhibited by Hotair knockdown. *P < 0.05; **P < 0.01.

On the other hand, the angiogenic effect of Hotair on NPC cells was evaluated. CNE1 and CNE2 cells were infected with Lv-ShHotair and the conditional medium was collected to incubate with HUVEC cells. A dramatically suppressive effect of tube formation was observed in HUVEC cells treated with the Lv-ShHotair condition medium (Figure 3A and Supplementary Figure 1A). The expression of the angiogenic growth factors vascular endothelial growth factor A (VEGFA) and Angiopoietin2 (Ang2) were evaluated and the results showed that they were down-regulated by Hotair knockdown at mRNA and protein levels in NPC cells (Figure 3B and Supplementary Figure 1B). The enzyme-linked immunosorbent assay (ELISA) indicated that the secretion of VEGFA and Ang2 were also reduced by Hotair knockdown in CNE1 cells (Figure 3C). We also investigated the expression of other angiogenic factors such as bFGF, Agn1, and the dominant receptors VEGFR2, Tie2, Tie1, and the results displayed no obvious change in these genes expression with LV-ShHotair treatment (Supplementary Figure S2).

To further evaluate the *in vivo* effect of Hotar-mediated-angiogenesis, a dorsal window chamber model was used. The Lv-ShHotair or Lv-ShNC infected CNE1 cells were injected beneath the fascia of nude mice in the center of the window. Compared with mice injected with Lv-ShNC infected CNE1 cells, the neovascular formation was significantly suppressed in those injected with Lv-ShHotair-infected-cells at day 7 after the surgery (Figure 3D), and the quantitative analyses were showed in Figure 3E. Taken together, we proposed that silencing of Hotair may attenuate tumorigenesis through anti-proliferation and anti-angiogenesis in NPC.

### Hotair promoted VEGFA transcription by directly targeting VEGFA promoter

**Figure 4:**
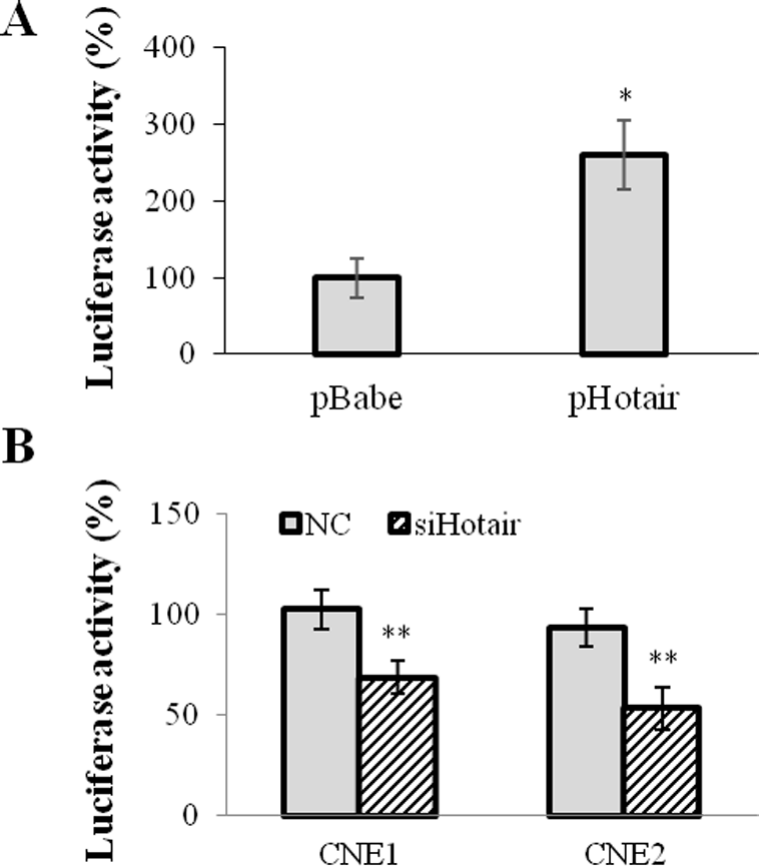
Hotair directly regulated VEGFA promoter activation 2.3 kb VEGFA promoter sequence was inserted into PGL3-enhancer vector and the luciferase activity was assayed. (**A**) Hotair overexpression significantly increased the luciferase activity of VEGFA promoter reporter in 293T cells. (**B**) Hotair knockdown profoundly repressed the luciferase reporter activity in NPC cells. *P < 0.05; **P < 0.01.

A 2.3kb VEGFA promoter (−2279∼+14) sequence was inserted into pGL3-enhancer vector and subsequently co-tranfected with Hotair expression vector to evaluate the transcriptional regulation of Hotair on VEGFA promoter. As shown in Figure 4, Hotair overexpression significantly increased in the luciferase activity of VEGFA promoter in 293T cells (Figure 4A), while siHotair suppressed its luciferase activity in CNE1 and CNE2 cells (Figure 4B).

For determination of HOTAIR-responsive elements sequence in the VEGFA promoter, the luciferase reporter vectors containing two halves of the promoter (−2279∼−1174 and −1174∼+14) were generated. The similar luciferase activity was seen in the −1174∼+14 sequence as that in the whole length sequence (2.3kb), while a low luciferase activity was shown in the −2279∼−1174 sequence. However neither overexpression nor knockdown of HOTAIR displayed significant effects on the luciferase activities of the luciferase reporters containing two previously mentioned fragments of the VEGFA promoter (Data not shown). Therefore, in terms of that lncRNAs regulate gene expression via recognizing specific DNA motif and then modifying genome structure, it suggests that both half sequences are crucial for the Hotair-mediated transcriptional activation of VEGFA promoter.

### GRP78 was identified as a functional target of Hotair in NPC cells

To identify the downstream molecular target of Hotair in NPC, a comparative proteomic profiling was performed. The representative pairs of silver-stained two-dimensional electrophoresis images were shown in Supplementary Figure 3A, and the cropped and enlarged sections of the paired spots were displayed in Supplementary Figure 3B. To further validate the results of 2-DE, the expression of 5 selected spots in the siHotair-treated CNE1 cells was examined by western blotting. A similar result was observed in the five selected proteins as that in proteomics profiling (Supplementary Figure 3C).

After comparing the two images, 52 protein spots were found to be differently expressed (at least a 3-fold change). Among these selected spots, a total of 43 proteins were successfully identified by MALDI-TOF MS and MS/MS analysis. 14 proteins (33%) were markedly up-regulated whereas 29 proteins (67%) were down-regulated in the siHotair-treated CNE1 cells. The characteristics of all identified proteins, including protein name, NCBI accession number, molecular mass/*pI*, protein score, fold change and function were all summarized in Supplementary Table S1. Among all the candidate proteins, GRP78 was one of the most significantly suppressed proteins after silencing of Hotair in CNE1 cells. 18.6-fold downregulation of GRP78 was displayed after knockdown of Hotair and a protein score of 109 in Mascot search indicated a highly significant match of GRP78 protein sequence.

### GRP78 was involved in Hotair mediated angiogenesis

**Figure 5:**
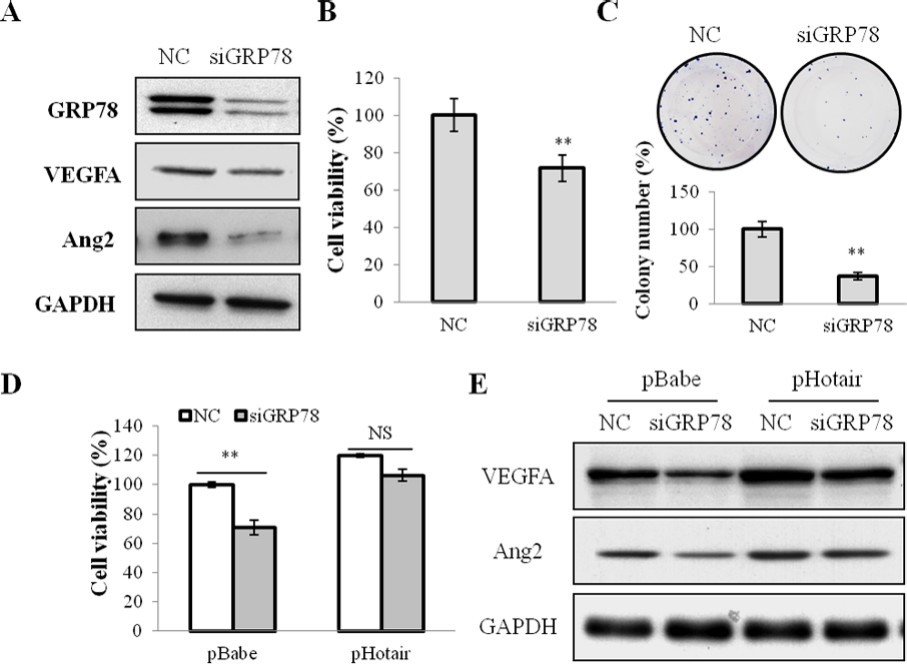
GRP78 knockdown suppressed cell viabilities and angiogenesis in NPC cells (**A**) the expression of GRP78, VEGFA and Ang2 was suppressed by siGRP78 in CNE1 cells. (**B** & **C**) the cell viability (B) and the colony formation (C) were inhibited by siGRP78 in CNE1 cells. (**D** & **E**) Hotair overexpression reversed the suppression of cell viability (D) and the suppressed expression of VEGFA and Agn2 (E) by siGRP78 in CNE1 cells. *P < 0.05; **P < 0.01.

To elucidate whether GRP78 was involved in Hotair-mediated-angiogenesis, we performed loss- and gain-of-function studies. Firstly, GRP78 expression was silenced by its specific siRNA (siGRP78) (Figure 5A). And its knockdown significantly attenuated cell growth (Figure 5B) and colony formation (Figure 5C), and the expression of VEGFA and Ang2 in CNE1 cells (Figure 5A). Notably, Hotair overexpression overcame the suppressive effect of siGRP78 on cell viabilities and angiogenesis in both CNE1 and CNE2 cells (Figure 5D and 5E and Supplementary Figure 4A).

**Figure 6:**
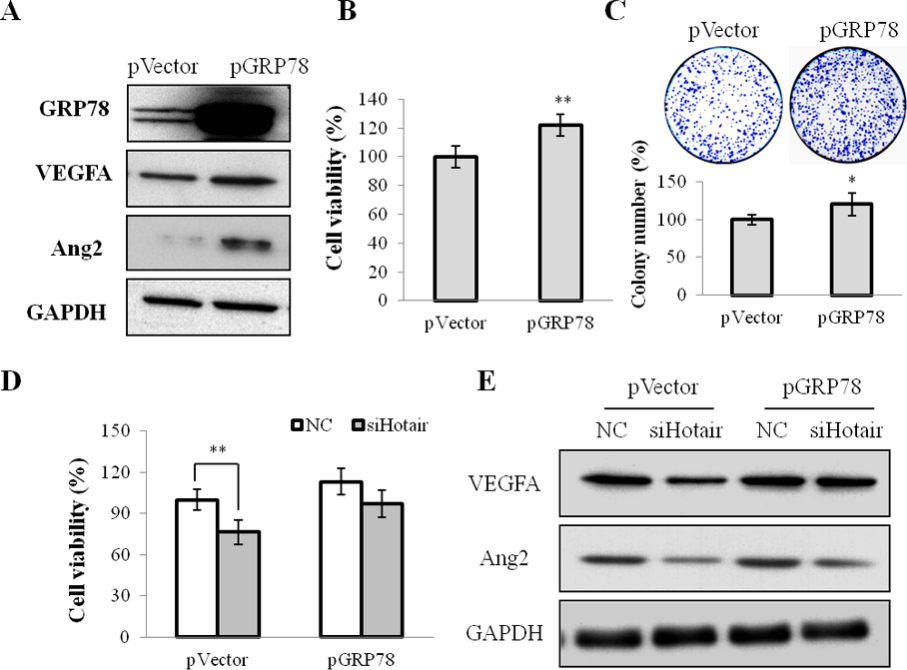
GRP78 overexpression mediated cell growth and angiogenesis in NPC cells (**A**) the expression of GRP78, VEGFA and Agn2 was up-regulated in CNE1 cells stably infected with pGRP78. (**B** & **C**) the cell viability (B) and the colony formation (C) were promoted by pGRP78 in CNE1 cells. (**D** & **E**) GRP78 overexpression rescued the siHotair-induced growth inhibition (D) and the siHotair-suppressed expression of VEGFA and Agn2 in CNE1 cells (E). *P < 0.05; **P < 0.01.

On the other hand, whether GRP78 overexpression could reverse the suppressive effect of Hotair in CNE1 cells was investigated. An expression vector pGRP78, encoding the full-length coding sequence of GRP78, stably restored its expression in CNE1 cells (Figure 6A). GRP78 overexpression promoted cell viability (Figure 6B) and stimulated colony formation (Figure 6C), and activated the angiogenic growth factors VEGFA and Ang2 expression (Figure 6A). Additionally, reinforced expression of GRP78 dramatically abrogated the siHotair-induced cell viability and angiogenesis inhibition in CNE1 and CNE2 cells (Figure 6D and 6E and Supplementary Figure 4B).

### Hotair knockdown reduced tumorigenicity through suppressing angiogenesis

**Figure 7:**
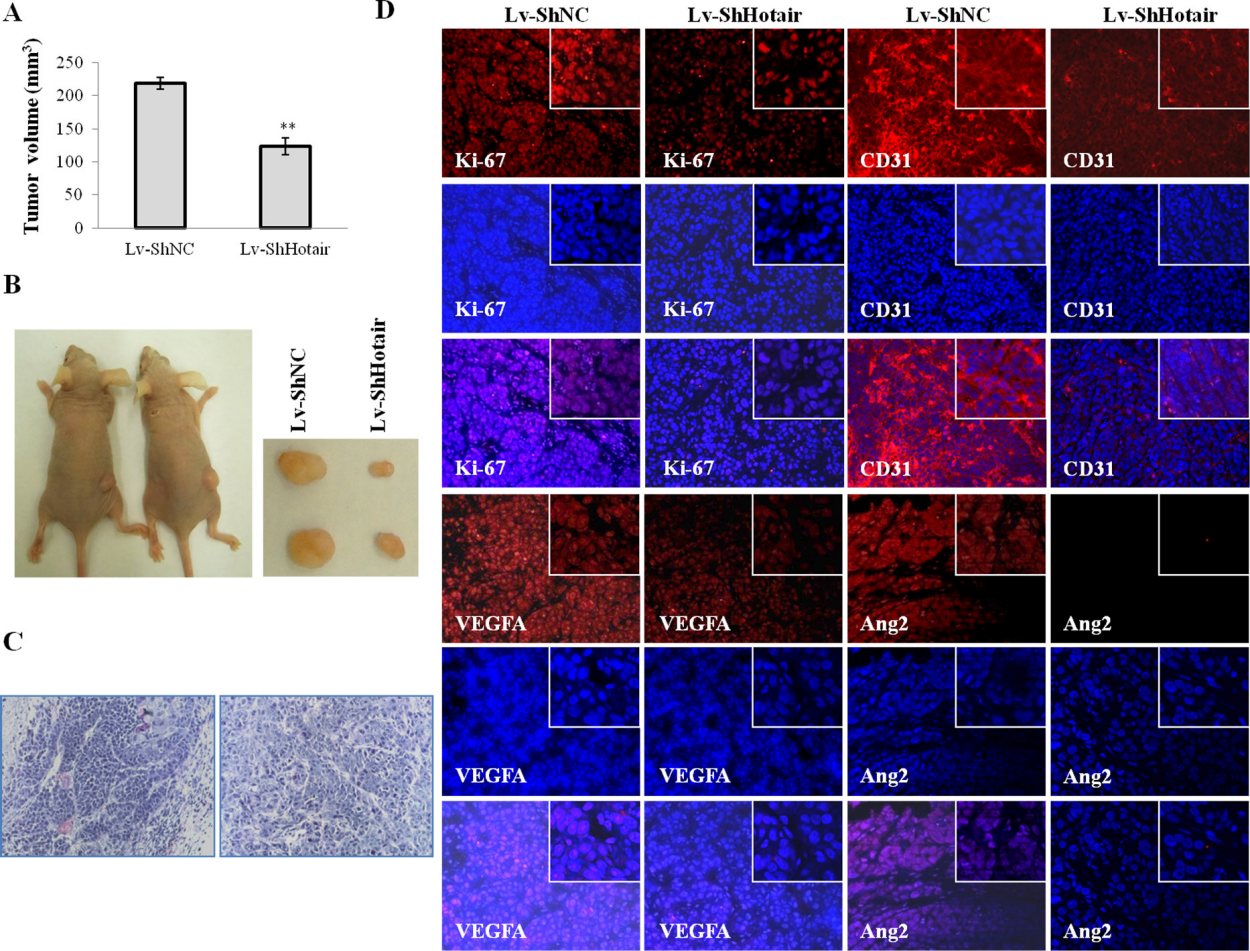
Hotair knockdown reduced tumorigenicity and angiogenesis in nude mice CNE1 cells were infected with Lv-ShHotair or Lv-ShNC and injected *s.c.* into nude mice. (**A** & **B**) Lv-ShHotair-infected cells generated smaller tumors than their control cells. The tumor volumes were measured and calculated (A) and the representative dissected tumor tissues from nude mice were showed (B). (**C**) (H & E) staining of the xenografts sections showed the microvessels inside the tumors were suppressed in Lv-ShHotair group. (**D**) the immunofluorescence staining of Ki-67, CD31, VEGFA and Ang2 in tumor sections. *P < 0.05; **P < 0.01.

Following the above observations, we further verified these *in vitro* findings by using an *in vivo* xenograft model. The CNE1 cells stably infected with Lv-ShHotair or Lv-ShNC were *s.c.* injected into the dorsal flank of nude mice. Compared with Lv-ShNC group, Lv-ShHotair group revealed a significant reduction in tumor volumes (Figure 7A) and sizes (Figure 7B). H & E staining displayed that the angiogenesis was suppressed in xenograft from Lv-ShHotair group (Figure 7C). Furthermore, decreased expression of the proliferation marker Ki-67, angiogenic marker CD31, VEGFA, Ang2 and GRP78 were observed in xenografts from the mice treated with Lv-ShHotair cells (Figure 7D and Supplementary Figure 5).

**Figure 8:**
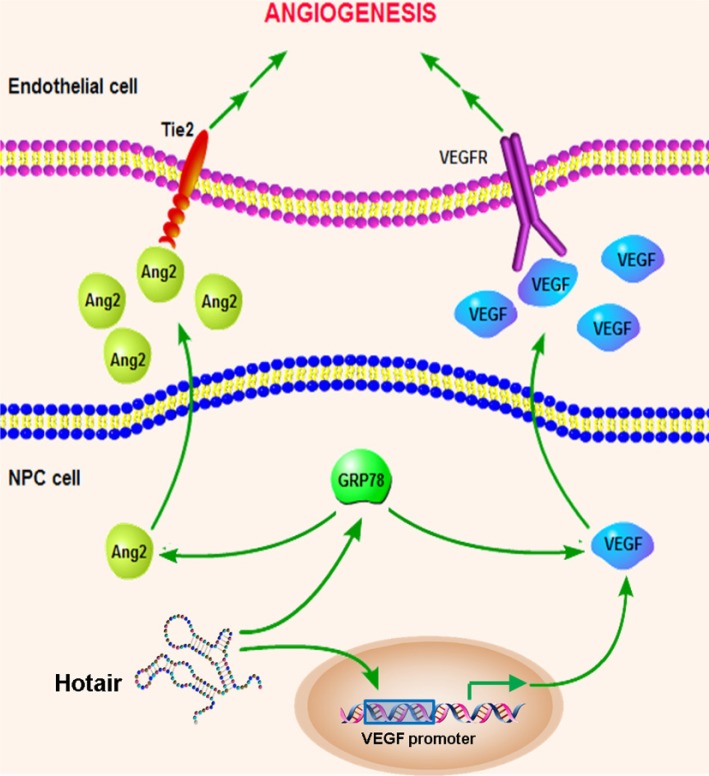
Schematic overview of the Hotair-mediated angiogenesis in NPC Hotair mediates angiogenesis through directly regulating VEGFA promoter transcriptional activity as well as activating VEGFA and Ang2 expression by up-regulating GRP78 expression.

Taken together, we pinpoint the underlying molecular mechanism of Hotair-mediated tumorigenesis that Hotair knockdown directly suppressed VEGFA transcriptional activation as well as reduced VEGFA and Ang2 expression by down-regulating GRP78 expression (Figure 8).

## DISCUSSION

Nasopharyngeal carcinoma is one of the most common cancers in Southern China and Southeast Asia. Its development might be associated with genetic factors [17]. In spite of encouraging advances in the molecular mechanism of NPC, the prognosis for patients with advanced NPC remains unfavourable. Hence, the molecular-targeted therapy will provide a more specific treatment for NPC and might provide new insight into its pathogenesis. In the present study, Hotair, a cancer-related lncRNA, was identified to contribute to the malignant tumor characteristics of NPC cells through involvement of cell proliferation and angiogenesis. To the best of our knowledge, this is the first time to report the novel function of Hotair on angiogenesis in cancer development.

As described above, Hotair initially was found to promote invasiveness and metastasis in a group of cancers [6–8]. Subsequent studies also documented that Hotair serves as a prognostic indicator for multiple cancers including NPC [16]. Our finding displayed that Hotair was especially up-regulated in NPC cell lines and tissues. As such, we hypothesize that Hotair mediates carcinogenesis of NPC. Herein, knockdown of Hotair suppressed cell viability and induced apoptosis of NPC cells. Moreover, it also negatively regulated *in vitro* colony formation as well as *in vivo* tumor growth.

Angiogenesis is a crucial step to the development of tumor in which the neovascularisation is indispensable to the formation of solid cancer [18]. On the other hand, anti-angiogenic agents impair the growth of tumor because of inadequate blood supply [19–20]. Therefore, anti-angiogenesis has been regarded as an attractive therapy to cancers. Among many growth factors that mediate angiogenesis, VEGF is known as the key regulator at the initial stage of tumor angiogenesis [21] and angiopoietin 2 (Ang2) plays a particular role in vessel maturation [22]. An anti-angiogenetic effect of Hotair knockdown was observed *in vitro* and *in vivo*. In addition, the expression and secretion of VEGFA and Ang2 was suppressed by Hotair knockdown in NPC cells and animal xenograft, which provides powerful evidence that silencing of Hotair acts as an anti-angiogenesis agent to NPC carcinogenesis.

After the function of Hotair in proliferation and angiogenesis was identified, elucidating the underlying mechanism became our research emphasis in the following studies. Hotair has been reported to actively participate in epigenetic modulation by recruiting PRC2 complex for promoter hypermethylation [6, 23–24]. Therefore, we first determined whether Hotair influences VEGFA promoter activity. The results of luciferase assays revealed that Hotair activated the transcription of 2.3 kb VEGFA promoter, but it has little effect on either half sequence (−2279∼−1174 and −1174∼+14) of the promoter, indicating that Hotair regulated VEGFA expression in length-dependent pattern. Given that lncRNA modulates gene expression by modifying the highly complicated chromatin structure, it is likely that the lengths of these two truncated VEGFA promoter fragments are not sufficient to form appropriate spatial structure, which is necessary for Hotair-mediated chromatin modification. Thus, we suppose that is why Hotair did not affect the luciferase activities of truncated VEGFA promoters.

Meanwhile, a functional proteomic profiling was performed in our study and 43 proteins were identified by using two-dimensional electrophoresis combined with MALDI-TOF/TOF MS and MS/MS analysis. Among them, 14 proteins were up-regulated and 29 proteins were down-regulated by siHotair in NPC cells. Particularly, GRP78 was validated to be a promising target for Hotair and an anti-cancer molecular target in NPC cells by using loss-of-function and gain-of-function studies, suggesting that it might be a novel molecular target of Hotair in NPC.

Glucose regulated protein 78 (GRP78), also known as the immunoglobulin heavy chain binding protein (BiP), belongs to the heat shock protein 70 (HSP70) family [25]. GRP78 was highly correlated with the tumor progression of various cancer types [26–29]. For example, it shows an anti-apoptotic and drug-resistant function in many cancer types such as colon cancer, breast cancer, and liver cancer [30–32]. Furthermore, GRP78 silencing suppressed the angiogenesis in colon cancer [33] and modulated tumor microenvironment during the tumor growth and metastasis [34]. Collectively, GRP78 can be a potent therapeutic target for anticancer therapy. In our study, GRP78 was demonstrated as an anti-angiogenetic target of Hotair in NPC cells. The knockdown of GRP78 could achieve a similar response as siHotair treatment and Hotair overexpression reversed the suppressive effect of siGRP78 on cell proliferation and angiogenesis. On the other hand, GRP78 overexpression rescued the siHotair-induced cell growth suppression in NPC cells. We therefore suggest that Hotair may function as a therapeutic candidate in NPC via suppressing GRP78 expression, and thus presents a significant promise for cancer therapy.

In summary, we identified the role of Hotair in mediating tumorigenesis and angiogenesis via directly promoting VEGFA transcription as well as activating VEGFA and Ang2 expression by up-regulating GRP78 expression. These results demonstrated that Hotair might serve as a promising diagnostic biomarker and therapeutic target for NPC. Therefore, disruption of the Hotair-mediated angiogenesis is highly promising for developing therapeutic strategies for NPC patients.

## MATERIALS AND METHODS

### Cell culture and clinical specimens

The human NPC cell lines CNE1, CNE2 and HONE1 cells were cultured in Dulbecco's modified Eagle's medium (DMEM) containing 10% fetal bovine serum and 1% penicillin-streptomycin, while the telomerase-immortalized nasopharyngeal epithelial cell line NP460 was maintained in a 1:1 mixture of refined KSFM medium (Invitrogen, Carlsbad, CA, USA) and Epilife (Sigma, St Louis, MO, USA).

20 clinical NPC samples and 5 non-tumor tissues were obtained from Sun Yat-Sen Memorial Hospital (Guangzhou, China) in accordance with the ethical standards of the institutional committee. Among them, 12 specimens were from male and 8 specimens were from female patients. At the collection, 12 patients were defined as stage III and 8 patients were classified at stage I and stage II (TNM classification).

### Transfection and quantitive real-time PCR analysis

The siHotair, siGRP78 and negative control (NC) were purchased from GenePharma (Shanghai, China). The Hotair overexpression plasmid (pHotair) was purchased from Addgene, while the plasmids pGRP78 and the control pVector were kindly provided by Dr. Ming-liang He (CUHK). Cells were seeded in 12-well plate and grew to 50% confluency for siRNA transfection and 90% for plasmid transfection. Plasmids and siRNAs were transfected with Lipofectamine 2000 (Invitrogen, USA) according to the manufacturer's protocol. At day 3 post-transfection, the cells were harvested and total RNA was extracted using TRIZOL reagent (Invitrogen, CA, USA). The total RNA was subjected to qRT-PCR analysis using the ImProm-II^™^ Reverse Transcription System (Promega, USA) and SYBR^®^ Premix Ex Taq^™^ II (TaKaRa). The primers for qRT-PCR were shown in Supplementary Table S2. All reactions were performed with a Step-One Plus Real Time PCR system (Applied Biosystems, USA) and done in triplicate.

### MTT and colony formation assays

The MTT and colony formation assays were performed for cell viability and proliferation determination. For MTT assay, 5 × 10^3^ cells per well were seeded in 96-well plate and incubated for 72 hours after transfection. 20 μL MTT per well was added and incubated for 3 hours. The medium was then removed and the crystals were dissolved in 100 μL DMSO. The absorbance at 570 nm was detected using Multiskan Go Microplate Readers (Thermo Fisher Scientific). For colony formation assay, cells were seeded into 6-well plate at a density of 5 × 10^2^ cells per well and cultured for another fortnight. The colonies were stained with Coomassie brilliant blue and counted under a microscope. All the experiments were performed in triplicate unless stated otherwise.

### Flow cytometric analysis

The cell apoptosis was determined by flow cytometric analysis. Cells (2 × 10^5^/well) were seeded in 6-well plate and transfected with NC or siHotair. 72 h after transfection, the apoptotic cells were quantified by a FITC-labelled AnnexinV/propidium iodide (PI) Apoptosis Detection Kit (Invitrogen), as described in previous report [35].

### The endothelial cell capillary-like tube formation assay

The capillary formation assay was performed to explore the effect of Hotair on *in vitro* angiogenesis. The 96-well plate was coated with matrigel and 2 × 10^4^ human umbilical vein endothelial cells (HUVECs) were seeded. The cells were then incubated in 100 μl condition medium that was harvested from CNE1/CNE2 cells transfected with Lv-ShNC or Lv-ShHotair. After 36 hours of incubation, the tubular structures network formation will be photographed [36].

### Establishment of hotair knockdown stable cell line

The Hotair knockdown stable cell line was constructed using shRNA lentiviral system. The plasmids carrying ShHotair or the negative control ShNC were co-transfected with packaging vectors pMDLg/pRRE, pRSV-REV and pCMV-VSVG to produce the pseudotyped lentiviruses designated as Lv-ShHotair and Lv-ShNC, as previously described [37]. The lentiviruses were concentrated by ultracentrifugation and then infected NPC cells with 8 μg/mL polybrene. Then, lentiviruses infected NPC were selected by puromycin (Sigma-Aldrich, USA) in the concentration of 0.5 μg/mL. After antibiotics selection for around 7 days, the cultured cells were collected and the knockdown of Hotair was confirmed by qRT-PCR.

### Intravital imaging of neovascularization in nude mice using dorsal window chamber model

4-week-old female nude mice were purchased from the Laboratory Animal Services Center of CUHK and randomly divided into two groups (*n* = 5). The dorsal skinfold window chambers (APJ Trading, USA) was surgically implanted onto the dorsal skin of nude mice as described by Palmer et al. [38]. Briefly, the dorsal skin of an anesthetized nude mouse was pulled up and a 1 cm circle was excised from the forward-facing surface of the skinfold. The hole was held with a pair of titanium frames. 5 × 10^5^ CNE1-ShHotair or CNE1-ShNC cells were injected beneath the fascia and the chamber was then covered with glass over slips. All mice were photographed and then euthanized on the seventh day following the operation when xenografts formed in the middle of the chamber.

### Luciferase reporter assay

The VEGFA promoter sequence (−2279∼+14) were amplified from human genomic DNA and subsequently inserted into the luciferase reporter vector pGL3-enhancer and designated as pGL3-VEGFA. The reporter plasmids were co-transfected with pHotair or control vector pBabe into 293T cells, or with siHotair or NC into CNE1 cells. After 28 hours incubation, the cells were lysed in reporter lysis buffer. The luciferase activities were measured by Luciferase Assay System (Promega, USA) and normalized to lysate protein concentration.

### Western blotting and Enzyme-Linked Immunosorbent Assay (ELISA)

The western blotting analysis was carried out as previous description^9^. The total cell lysates were extracted using RIPA buffer. The proteins were separated by 12% sodium dodecyl sulphate polyacrylamide gel electrophoresis (SDS-PAGE) and transferred to PVDF membranes (Millipore, Billerica, MA), which were then blocked with 5% skimmed milk and incubated with primary antibodies at 4°C overnight. Subsequently, the membranes were incubated with HRP-conjugated secondary antibodiesand finally detected with ECL substrate kit. The antibodies anti-Angiopoietin 2 (Ang2), anti-GRP78 were purchased from CST, USA, while anti-VEGFA, anti-eEF2, anti-Ezrin, anti-PRDX-1, anti-Keratin-8 and anti-GAPDH were purchased from Santa Cruz.

Secretion of VEGFA and Ang2 by CNE1 cells was evaluated by ELISA kits purchased from Dakewe Biotech (Beijing, China) and Cloud-Clone Corp (USA), respectively. The CNE1-ShHotair and CNE1-ShNC cells were seeded into 12-well plate and cultured for 24 hours. The supernatants were collected and the ELISA assays were performed according to the manufacturer's protocol.

### Proteomic profiling using two-dimensional electrophoresis (2-DE) and MALDI-TOF MS analysis

For 2-DE analysis, protein samples (150 mg) were diluted in 250 mL rehydration buffer (8 M urea, 2% CHAPS, 0.4% DTT, 0.5% IPG buffer, 0.002% bromophenol blue). The two dimension of isoelectric focus (IEF) and SDS-PAGE was performed as described previously^39^. After the electrophoresis, the gels were stained silver nitrate. The protein spots of interest were excised from the gel and digested by trypsin. The mass fingerprinting of hydrolysed peptides by MALDI-TOF MS and MS/MS was carried out and analyzed in Li Ka Shing Institute of Health Sciences, CUHK [39].

### Tumor xenograft model

1 × 10^6^ CNE1-ShNC or CNE1-ShHotair cells were injected into the dorsal flanks of 4-week-old female nude mice, respectively. The diameters and the growth of tumor xenografts were investigated. The xenografts size was measured every 3 days according to the formula: volume = 1/2 (shortest diameter)^2^ × (longest diameter). When the tumors grew to 500 mm^3^, mice were sacrificed and the xenografts were dissected and weighed.

### Immunofluorescence staining and hematoxylin-eosin (H & E) staining

Histology studies were carried out as described previously [9, 40]. The dissected tumors were fixed overnight in 4% paraformaldehyde, embedded in paraffin and then sectioned at 5 μm. The sections mounted on the glass slides were deparaffinized and rehydrated. The counterstaining with hematoxylin and eosin were performed following the standard protocol. For immunofluorescence assay, the sections were incubated with antibodies for proliferation marker Ki-67 (Calbiochem, Cambridge, MA), endothelial cell marker CD31 (Santa Cruz, CA), VEGFA, GRP78 and Ang2, followed by incubation with Alexa Fluor^®^ conjugated secondary antibodies. After the cell nuclei were labeled with DAPI, images were captured by Zeiss Axiophot 2 microscope.

### Statistical analysis

Data are expressed as mean ± SD. Statistical analysis was performed using the two-tailed Student's *t* test. A *p* value less than 0.05 is considered to be statistically significant.

## SUPPLEMENTARY MATERIALS FIGURES AND TABLES


