# Supplementary material for: Long noncoding RNA hotair mediated angiogenesis in nasopharyngeal carcinoma by direct and indirect signaling pathways
Source: Oncotarget. 2015 Dec 22;7(4):4712–23. doi: 10.18632/oncotarget.6731 (PMC4826237; doi:10.18632/oncotarget.6731)
Supplement: Supplementary file 1 [file oncotarget-07-4712-s001.pdf]

Long noncoding RNA hotair mediated angiogenesis in nasopharyngeal carcinoma by direct and indirect signaling pathways

Supplementary Materials

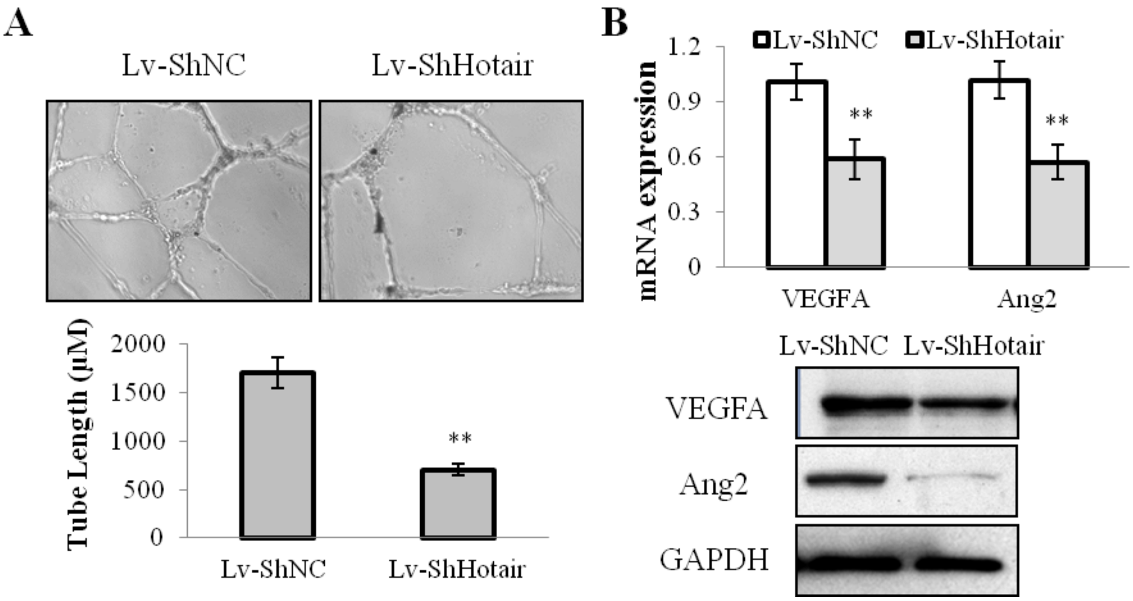

Supplementary Figure S1: Hotair mediated angiogenesis in CNE2 cells. (**A**) the ability of endothelial cell tube formation was suppressed in the Lv-ShHotair-infected condition medium, as compared to the Lv-ShNC infected condition medium. (**B**) the angiogenic growth factors VEGFA and Ang2 were down-regulated in the Lv-ShHotair infected CNE2 cells at mRNA and protein levels. *$P < 0.05$; **$P < 0.01$.

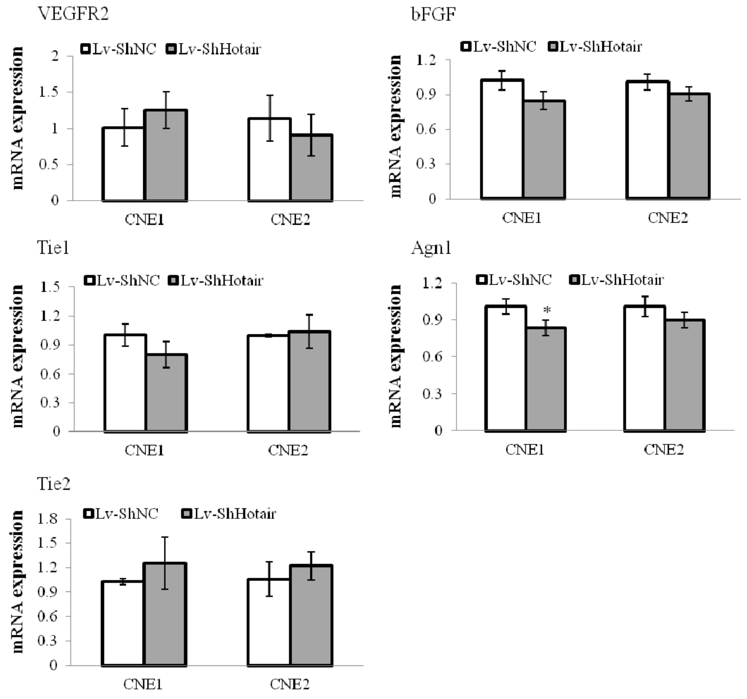

Supplementary Figure S2: The expression of other angiogenic factors was not significantly affected at mRNA level by Hotair knockdown.

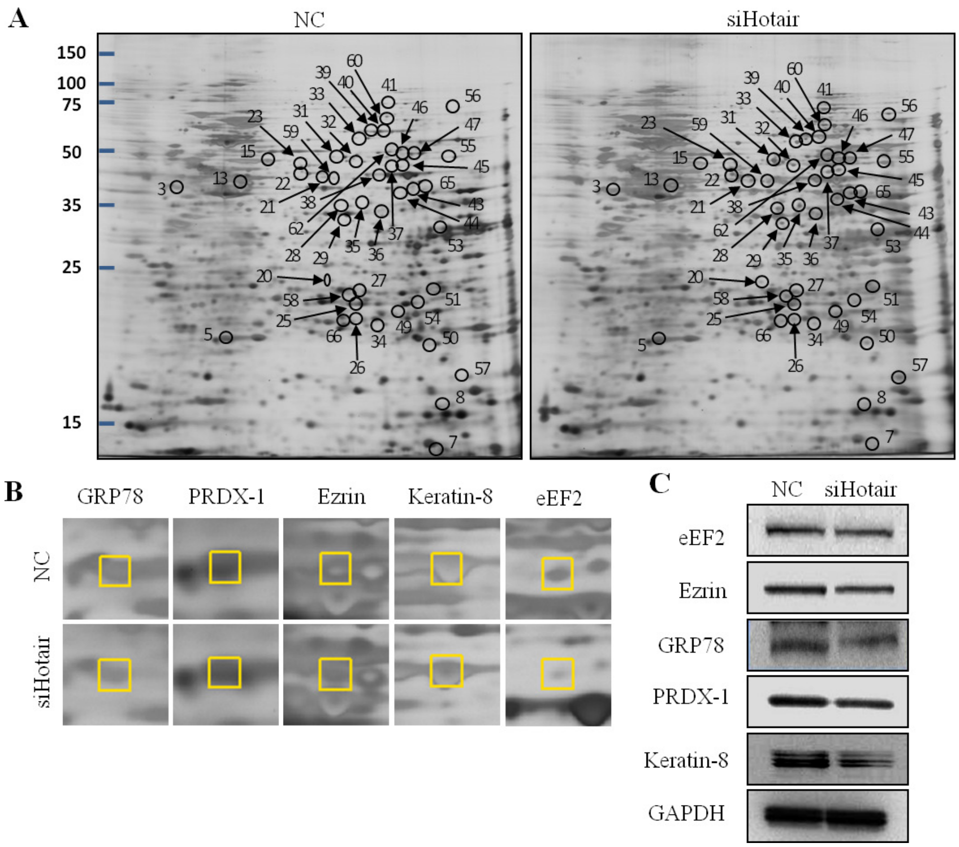

Supplementary Figure S3: GRP78 was a direct target of Hotair in NPC cells. (**A**) the representative silver-stained 2-DE images of CNE1 cells treated with siHotair or NC by using proteomic profiling ($n = 3$). The protein of spot 3 was identified to be human GRP78 by protein database search. (**B**) enlarged images of differentially expressed protein in the two groups. (**C**) the five proteins were confirmed by using Western blotting. *$P < 0.05$; **$P < 0.01$.

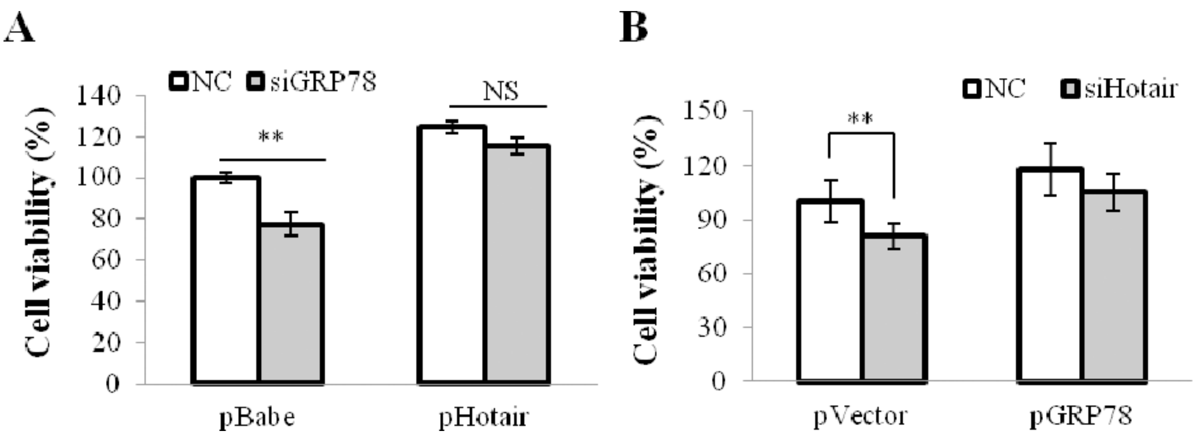

Supplementary Figure S4: GRP78 mediated cell growth in CNE2 cells. (**A**) Hotair overexpression reversed the suppression of cell viability by siGRP78. (**B**) GRP78 overexpression rescued the siHotair-induced growth inhibition. *$P < 0.05$; **$P < 0.01$.

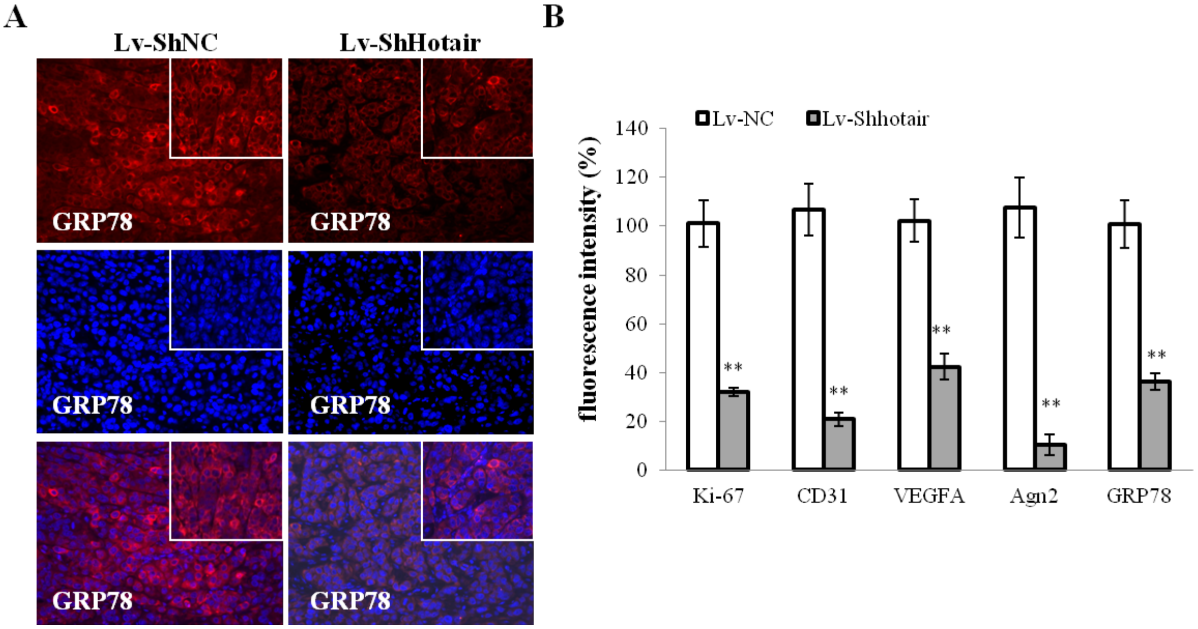

Supplementary Figure S5: The immunofluorescence double staining analyses. (**A**) GRP78 expression was suppressed in Lv-ShHotair treated group. (**B**) the quantitative analyses for the immunofluorecence staining of Ki-67, CD31, VEGFA, Agn2 and GRP78. *$P < 0.05$; **$P < 0.01$.

Supplementary Table S1: Differentially expressed proteins identified by 2-DE and MS analysis between siHotair-treated and NC-treated CNE1 cells

Spots No.	Accession No.	Protein name	MW	PI	Score	change fold
Cytoskeleton						
49	Q12955	Ankyrin-3 (ANK3)	482394	6.07	61	(+)
36	P11532	Dystrophin	428479	5.65	58	3.4x ↓
13	P15311	Ezrin	69484	5.94	66	27.2x ↓
7	P07737	Profilin-1	15216	8.44	122	(+)
15	P05787	Keratin, type II cytoskeletal 8	53671	5.52	165	30x ↓
62	P13647	Keratin, type II cytoskeletal 5	62568	7.59	78	(−)
39	P02545	Lamin-A/C	74380	6.57	244	3.4x ↓
RNA processing and modification						
58	P31942	Heterogeneous nuclear ribonucleoprotein H3	36960	6.37	151	(−)
65	Q14103	Heterogeneous nuclear ribonucleoprotein D0	38581	7.62	127	(−)
53	P22626	Heterogeneous nuclear ribonucleoproteins A2/B1	37464	8.97	70	4.2x ↑
Regulation of transcription and translation						
45	P13639	Elongation factor 2	96246	6.41	70	4.3x ↓
41	Q92945	Far upstream element-binding protein 2	73443	6.84	123	3.9x ↓
60	Q96AE4	Far upstream element-binding protein 1	67690	7.18	103	(−)
35	Q15365	Poly(rC)-binding protein 1	37987	6.66	62	3.2x ↓
32	Q9Y265	RuvB-like 1	50538	6.02	80	4.5x ↓
Post-translational modification						
29	A2A3K4	Protein tyrosine phosphatase domain-containing protein 1	85543	7.35	109	3.3x ↓
21	O75439	Mitochondrial-processing peptidase subunit beta	55073	6.38	88	3.5x ↓

Chaperones and stress response						
3	P11021	78 kDa glucose-regulated protein	72402	5.07	109	18.6x ↓
51	P11142	Heat shock cognate 71 kDa protein	71082	5.37	358	3.0x ↑
5	Q06830	Peroxiredoxin-1	22324	8.27	61	7.6x ↓
25	P30041	Peroxiredoxin-6	25133	6	67	3.4x ↑
66	P61758	Prefoldin subunit 3	22815	6.63	60	(+)
33	P40227	T-complex protein 1 subunit zeta	58444	6.23	105	5.7x ↓
31	P78371	T-complex protein 1 subunit beta	57794	6.01	157	3.8x ↓
Chaperone regulators						
26	O95816	BAG family molecular chaperone regulator 2	23928	6.25	68	3.2x ↑
Metabolic enzymes and regualtors						
38	P06733	Alpha-enolase (ENO1)	47481	7.01	78	6.0x ↓
55	P25705	ATP synthase subunit alpha, mitochondrial	59828	9.16	358	3.4x ↑
43	P22695	Cytochrome b-c1 complex subunit 2, mitochondrial	48584	8.74	56	3.9x ↓
52	P04406	Glyceraldehyde-3-phosphate dehydrogenase (GAPDH)	36201	8.57	122	6.0x ↑
57	P51970	NADH dehydrogenase [ubiquinone] 1 alpha subcomplex subunit 8	20548	7.57	34	3.9x ↓
8	P22392	Nucleoside diphosphate kinase B	17401	8.52	83	(+)
44	P00558	Phosphoglycerate kinase 1	44985	8.3	74	4.2x ↓
27	P18669	Phosphoglycerate mutase 1	28900	6.67	106	4.7x ↓
22	P14618	Pyruvate kinase isozymes M1/M2	58470	7.96	220	21.5x ↓
46	Q9NR45	Sialic acid synthase	40738	6.29	55	5.2x ↑
54	P60174	Triosephosphate isomerase	26938	6.45	82	(+)
41	P53007	Tricarboxylate transport protein, mitochondrial	34333	9.91	63	3.9x ↓

Growth regulation						
56	O60566	Mitotic checkpoint serine/ threonine-protein kinase BUB1 beta	120781	5.2	69	4.2x ↓
59	Q9UQ80	Proliferation-associated protein 2G4	44101	6.13	50	(−)
40	Q9BXU1	Serine/threonine-protein kinase 31	116647	5.04	56	47x ↓
Miscellaneous functional proteins						
52	P07355	Annexin A2	38808	7.57	72	6.0x ↑
50	P30086	Phosphatidylethanolamine-binding protein 1	21158	7.01	178	3.6x ↑
23	Q9H0H3	Ectoderm-neural cortex protein 2	66964	6.1	60	3.7x ↓

With a 2-fold difference cutoff in intensity, 52 spots were selected for further MALDI-TOF-MS/MS-MS analysis, and 43 proteins were finally identified. CI, confidence interval

Supplementary Table S2: Primers for the qPCR analysis

Gene	Primer sequence (5′-3′)
GAPDH	*Forward:* TATTCAGGAAGGTGTTACTTAA
	Reverse: TAAGGCACCCTTCTGAGTAGA
Hotair	*Forward:* CAGTGGGGAACTCTGACTCG
	Reverse: GTGCCTGGTGCTCTCTTACC
VEGFA	*Forward:* CTACCTCCACCATGCCAAGT
	Reverse: GCGCTGCGCTGATAGAAGTA
Ang2	*Forward:* CTGGGAAGGGAATGAGGCTT
	Reverse: CATGCATCAAACCACCAGCC
VEGFR2	*Forward:* TGATGTGGTTCTGAGTCCGT
	Reverse: AGACTGGGTTTTTAGGTCTCGG
Tie-1	*Forward:* TGGCTGCTCTTGTGGATCTG
	Reverse: TCTCACAGTGCACTCCATGC
Tie-2	*Forward:* TTCACCAGGCTGATAGTCCG
	Reverse: ACACGTCCTTCCCATAAACCC
